# Editorial Notes

**Published:** 1883-12

**Authors:** 


### Editorial Notes.

The Chicago correspondent of the Philadelphia *Medical Times* sends the following from the metropolis of the West:

"It is becoming fashionable in this city to advertise the operators and operations in surgery. Some invite the newspaper reporter to be present, others see to it that the paper is supplied with an entirely technical report of the case, and others go so far as to secure an announcement in advance, to be followed by an exhaustive description of the operation in which the usual statements are made to the effect that the operation has been performed but twice before and never till this time in this country. They usually close with the assurance that the *operator* and the patient are doing well. Last week the popular mind was considerably agitated in this manner over the ligation of the common carotid."

AFTER an absence of six months, occupied in traveling in Europe and the South, Dr. Van Peyma returns to assume, with this number of the JOURNAL, his share of editorial control and responsibility.

OLEATES. We call attention to the value of these new preparations in the treatment especially of cutaneous diseases. We have used those prepared by Parke, Davis & Co. and Schieffelin, with great satisfaction. Messrs. Shaver & Co. inform us that they keep in stock a full supply and also have received a large invoice of the true "Sapo Viridis" and "Liquor Carbonis Detergens," so highly commended in Duhring's and other works on skin diseases.

